# Deposition of Selective Catalytic Reduction Coating on Wire-Mesh Structure by Atmospheric Plasma Spraying

**DOI:** 10.3390/ma12183046

**Published:** 2019-09-19

**Authors:** Xiaoyu Ma, Yunlong Ma, Hui Li, Yingliang Tian

**Affiliations:** College of Materials Science and Engineering, Beijing University of Technology, Beijing 100124, China; maxiaoyu@bjut.edu.cn (X.M.); ddt753@sina.cn (Y.M.); tianyl@bjut.edu.cn (Y.T.)

**Keywords:** atmospheric plasma spray, wire-mesh structure, selective catalytic reduction, Mn-Ce-Ti mixed oxides

## Abstract

A series of catalytic coatings consisting of MnO_x_-CeO_2_ and TiO_2_ support were prepared by atmospheric plasma spraying, which was aimed at the application of selective catalytic reduction (SCR) of NO_x_. The effect of the load of active component on the coating was firstly studied. The results showed that all the coating presented the highest catalytic activity at approximately 350 °C and the coating with the composition of 20MnO_x_/5CeO_2_/TiO_2_ (wt%) achieved the most powerful performance. The coating was then prepared on a wire-mesh structure substrate, which can be easily assembled as a gas filter. The results showed that the specific surface area was greatly increased resulting in the significant improvement of the catalytic activity of the coating. This strategy offered a promising possibility of removing NO_x_ and particulate fliting simultaneously in industrial applications.

## 1. Introduction

Nitrogen oxides (NO_x_) are the main pollutants in the air, which are emitted by vehicle exhaust, combustion of fossil fuels, especially from electric power plants. The NO_x_ is very harmful to the environment, which results in acid rain, photochemical smog, and ozone hole [1,2]. The selective catalytic reduction (SCR) with NH_3_ is currently the most widely used measures that transform NO_x_ emissions from stationary sources to N_2_ and H_2_O, primarily through the reaction [3]:

4NO + 4NH_3_ + O_2_ = 4N_2_ + 6H_2_O (1)

In general, the commercially used catalyst for the SCR process is V_2_O_5_/TiO_2_ (anatase) catalyst, which can be enhanced by the addition of WO_3_ or MoO_3_ [3,4,5,6,7]. However, this kind of catalyst only has an excellent efficiency at an elevated temperature range (300–400 °C) and will be easily disable in the environment containing SO_2_. In this context, the Mn-based catalysts was found to be an excellent candidate in the SCR processing in low temperature, which could be located at the downstream of the desulfurization and particulate control devices. The contact with SO_2_ and small particulates could be reduced [8]. Specifically, the MnO_x_-CeO_2_ supported by anatase TiO_2_ offers high catalytic activity at a relatively lower temperature [3,8,9,10]. The CeO_2_ transforms to Ce_2_O_3_ in the reaction owing to its large oxygen storage capacity. Since the TiO_2_ (anatase) has a large surface area and it provides more active sites, it is an excellent support in the catalyst for NO_x_ removal. Moreover, the MnO_x_ can be better dispersed on the TiO_2_ support, and a fine synergistic effect could be generated between MnO_x_ and anatase [11].

Currently, many works on SCR performances were tested in the laboratory, and the majority of these works concerned the powder-based catalysts only, the reports on the coating-type catalysts that can be applied on a large scale was rarely found. One relevant work on washcoat-type catalysts were prepared on the honeycomb cordierite (CC) substrate [12]. However, these monoliths with narrow channel structures inside yield significant air pressure drop, in which a laminar gas flow takes place so that they had a low heat-transfer and mass-transfer efficiency. Using wire-mesh as substrate could overcome this circumstance to a large extent, because it has greater heat transfer numbers, a moderate pressure drop, good thermal and mechanical strength, and an excellent thermal response as well due to the porous structure [13,14,15]. The air pressure drop could reach 4000 Pa in cordierite, which is much higher than the porous wire-mesh (no more than 1000 Pa) [16]. Additionally, the heat-transfer and mass-transfer coefficient was influenced by the wire-mesh thickness and the variable channel radius. Moreover, the wire-mesh typed is much cheaper, whose coast is about only 25% of the ceramic honeycombs and foams. Furthermore, the high geometric flexibility of wire-mesh can meet many different shape requirements in actual applications. These features make it easy to be used in the manufacturing of gas filter or gas heater, which can remove NO_x_ and eliminate dust simultaneously.

Atmospheric plasma spray (APS) is a low cost and high flexibility deposition technique in the industry, which could produce a variety of coatings on any substrate. Moreover, the ceramic coating prepared by APS with porous structure could increase the specific area of the surface, therefore leading to an enhancement of the catalytic reaction [17,18]. Coating deposited on the substrate by plasma spraying instead of the common wet-dipping approach also gives a better adhesion of coating [19]. Due to the fully-molten state and semi-molten state of the feedstock by plasma spraying deposited on the surface, the coating could naturally have a well adhesion and achieve a longer service life.

In this work, MnO_x_-CeO_2_-TiO_2_ powder with different composition was firstly prepared as the feedstock. The composition of the coating was optimized in terms of deNO_x_ efficiency. The coating was then prepared on a stainless-steel plate and wire-mesh substrate by plasma spraying, respectively. The coating’s catalytic performance on both substrates was examined and compared.

## 2. Materials and Methods

### 2.1. Feedstock Preparation

**Figure 1:**
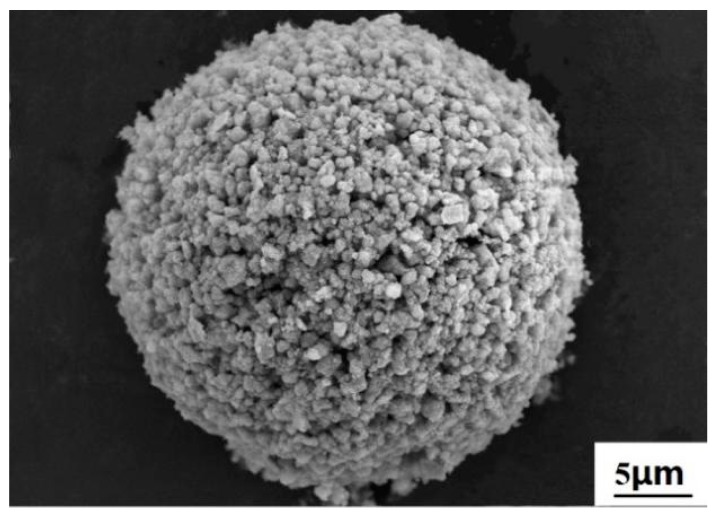
The morphology of agglomerated powder.

The initial raw powder included anatase TiO_2_ with a size of 10 nm, MnO_x_ of 50 nm, and CeO2 of 50 nm (99.9%, Tansail, Nanjing, China). Since the nano-size powders are easily agglomerated and can hardly be transported by a flowing gas, deposition of the nano-size particles by thermal spraying usually required that the feedstock is in micro-size particles. To this end, the composite powder was firstly mixed with deionized water, a small amount of binder (PVA) and dispersant (PAA) was then added to the slurry. After three h of ball milling, the slurry was transferred to an atomization spray-dryer to agglomerate the nano-size powder. Finally, the agglomerated particles size ranging from 20–40 μm with a spherical shape was obtained, as shown in Figure 1. This agglomeration process just involved morphological changes and no phase change was taken place during the spray-drying [20].

### 2.2. Coating Preparation

**Figure 2:**
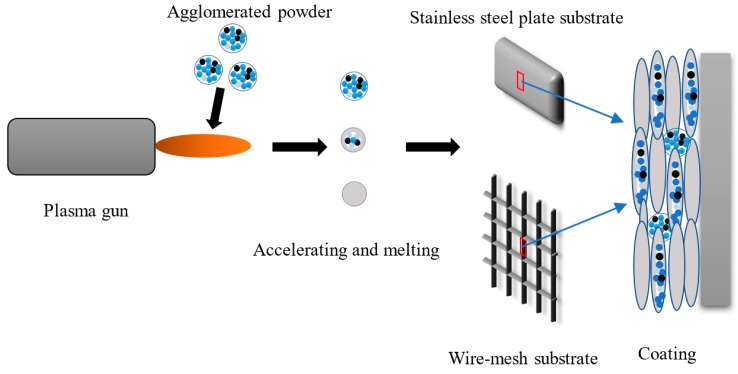
Schematic diagram of the process of plasma spraying.

Two types of substrates were used, one was a stainless-steel plate with a size of 60 mm × 15 mm × 4 mm, the other was a wire-mesh substrate made of stainless wire with a diameter of 0.25 mm and the grid cell size was of 0.5 mm. The schematic diagram of the spraying process is shown in Figure 2. Prior to spraying, the plate substrate surface was cleaned by ethanol and then roughened by sand-blasting treatment with 16 to 22 mesh corundum, as usual. In the case of wire-mesh substrate, it is difficult to perform a sand-blast pre-treatment. Therefore, the NiAl coating was sprayed on the wire-mesh firstly as a bond layer, which offered an adequate adhesion of the catalytic coating [19]. After that, the coating was deposited using Metco 9 MB plasma gun mounted a robot arm. The spraying parameter of the catalytic coating as well as the NiAl are given in Table 1. During the plasma spraying process, the spraying time and the track of the plasma gun was controlled to maintain the coating thickness and uniformity. The speed of the robot is 500 mm/s and the deposited coating thickness for each cycle is approximate 10 μm. In addition, both the front and back of the substrate was sprayed.

### 2.3. Characterization

The phase of the coatings was examined by an X-ray detector (Shimadzu, Xrd-7000, Kyoto, Japan) in which the Cu target was used (Kα radiation) and the scanning range was from 20° to 60° at a scanning speed 0.02°/s. In order to calculate the fraction of anatase phase in the coating, the Jade 6.0 software was used. The full-spectrum of the diffraction peak was firstly fitted and then the anatase content was estimated by calculating the diffraction peak area, the equation was shown as follows:

(2) $$A=\frac{I_{anatse}}{I_{total}}$$

A: The fraction of anatase phase in the coating
*I_anatase_*: The area of anatase
*I_total_*: The total area of the diffraction peak area

In order to observe the coating microstructure, the coating samples were firstly sectioned by electrical discharge machining (EDM), and then cold mounted with epoxy resin. The sample was subsequently grinded and polished. The cross-section of the coating sample was observed by SEM. The elements analysis on different area was also performed by the SEM equipped energy dispersive spectrometer (EDS).

**Figure 3:**
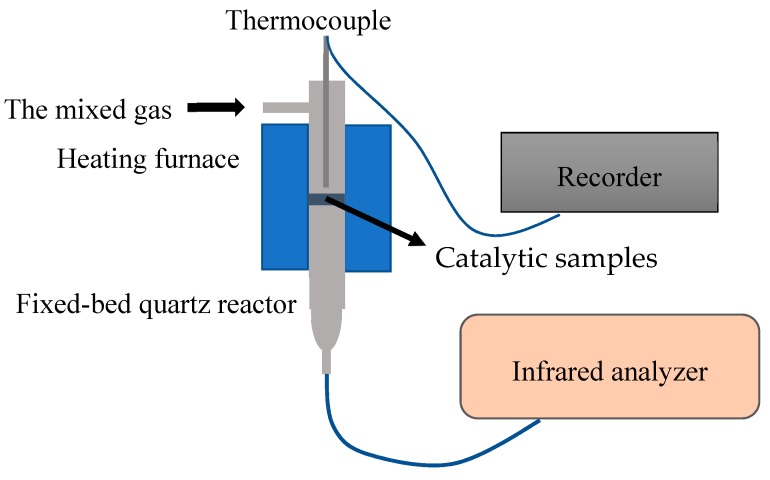
Schematic diagram of the selective catalytic reduction (SCR) testing reactor.

The SCR activity test was carried out in a fixed-bed quartz reactor. The schematic diagram of the SCR reactor is shown in Figure 3. In which a vertical tube furnace was used to heat the mixed gas. The typical gas composition was as follows: 1000 ppm NH_3_, 1000 ppm NO, 5% O_2,_ and the balance of N_2_. The total flow rate was 1350 mL/min and the hourly space velocity (GHSV) was 27,000 h^−1^. The 42i-HL NO/NO_x_ analyzer was used to monitor the concentration of NO_x_ in different temperature. Before the data recording, the reaction gas was purged into the reactor until the NO concentration of the export gas reached the inlet gas concentration (1000 ppm). The data was then recorded for every 50 °C and the NO_x_ conversion efficiency and N_2_ selectivity was calculated by the following equation [21,22].

(3) $$\eta_{NO_{x}}=({\left[NO_{x}\right]}_{in}-{\left[NO_{x}\right]}_{out}/{\left[NO_{x}\right]}_{in})\times 100\%\;\left[NO_{x}\right]=\left[\mathrm{NO}\right]+\left[N_{2}O\right]+\left[NO_{2}\right]$$

(4) $$N_{2}\;selectivity=\frac{{\left[NO_{x}\right]}_{in}-{\left[NO_{x}\right]}_{out}-{\left[NO_{2}\right]}_{out}-{\left[N_{2}O\right]}_{out}}{{\left[NO_{x}\right]}_{in}-{\left[NO_{x}\right]}_{out}\;}\times 100\%$$

## 3. Results and Discussion

### 3.1. Coatings Deposited on Steel Plate

**Figure 4:**
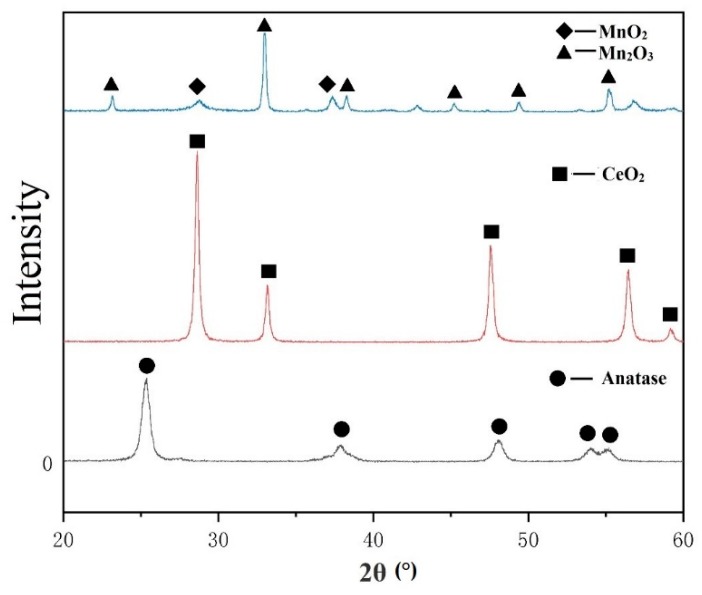
XRD of the initial powder.

**Figure 5:**
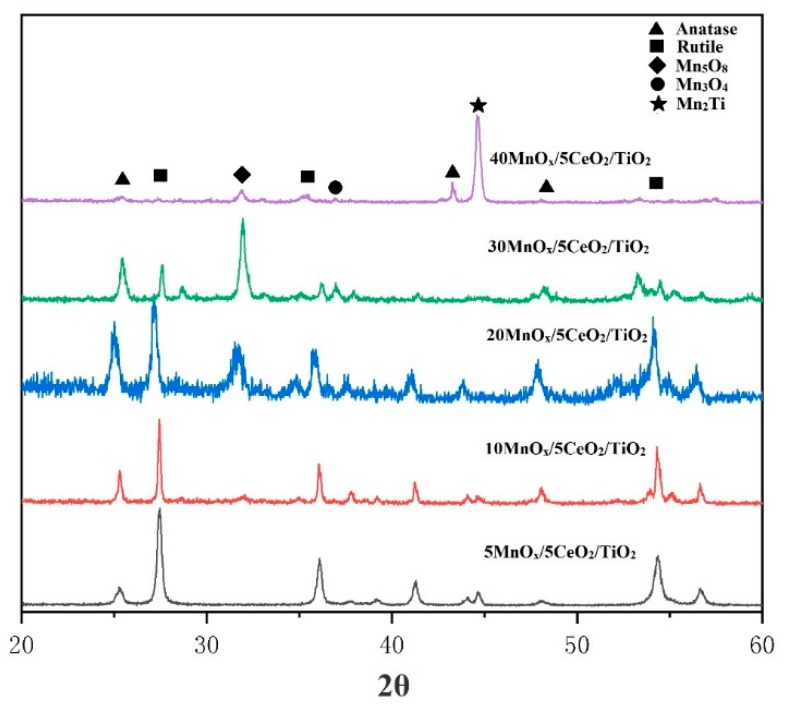
XRD pattern of plasma sprayed coating on steel plate with different composition.

The XRD pattern of the powder is shown in Figure 4. It was shown that all the original TiO_2_ was in anatase, while MnO_x_ was composed of MnO_2_ and Mn_2_O_3_. In general, anatase would transfer to rutile at the temperature of 400–1100 °C [23]. In the process of plasma spraying, the powder was usually in a molten or semi-melted state, thus rutile was readily formed after spraying. The XRD pattern of the coatings with different constitution is shown in Figure 5. The content of anatase in each coating is given in Table 2. It was shown that under the same spraying parameters, minor retention of anatase phase occurred for 5MnO_x_/5CeO_2_/TiO_2_ coating, in which most of anatase was transformed to rutile. As the content of MnO_x_ increased to 10% and 20%, although the total amount of TiO_2_ decreased, the content of anatase phase in the coating increased obviously. The melting point of Mn_2_O_3_ is 1080 °C, which is much lower than that of TiO2 (1600 °C). During the heating journey of the in-flight particles in the plasma jet, MnO_x_ could absorb the heat and then be melted earlier than the melting of TiO_2_, which likely counteracted the phase transformation of anatase to rutile. This result also gives a hint that the addition of MnO_x_ with low-melting point influenced the phase change of TiO_2_ during plasma spraying. As the amount of MnO_x_ increased to 30%, the original content of TiO_2_ decreased to 65%, and the intensity of anatase in the coating was reduced. When the content of MnO_x_ reached 40%, there was only little anatase and rutile survived in the coating. Moreover, the phase of MnO_x_ also underwent phase change, the XRD results indicated that as the fraction of MnO_x_ increased from 5% to 30%, the metastable Mn_5_O_8_ and Mn_3_O_4_ was formed in the coating. When the initial content of MnO_x_ increased to 40%, the content of Mn_5_O_8_ and Mn_3_O_4_ in the coating decreased, but Mn_2_Ti phase was produced in the coating, which might result from the interaction of MnO_x_ and TiO_2_.

### 3.2. Morphology and Composition Analysis of Coatings Deposited on Steel Plate

**Figure 6:**
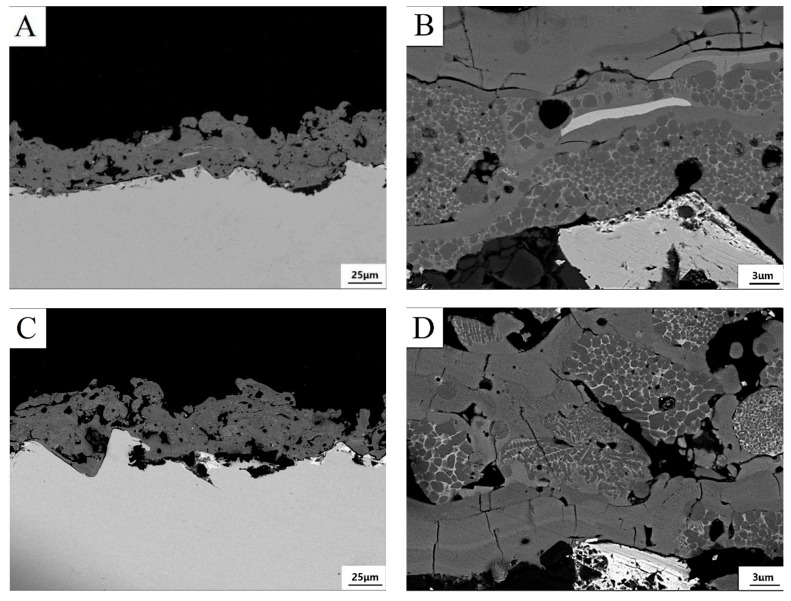

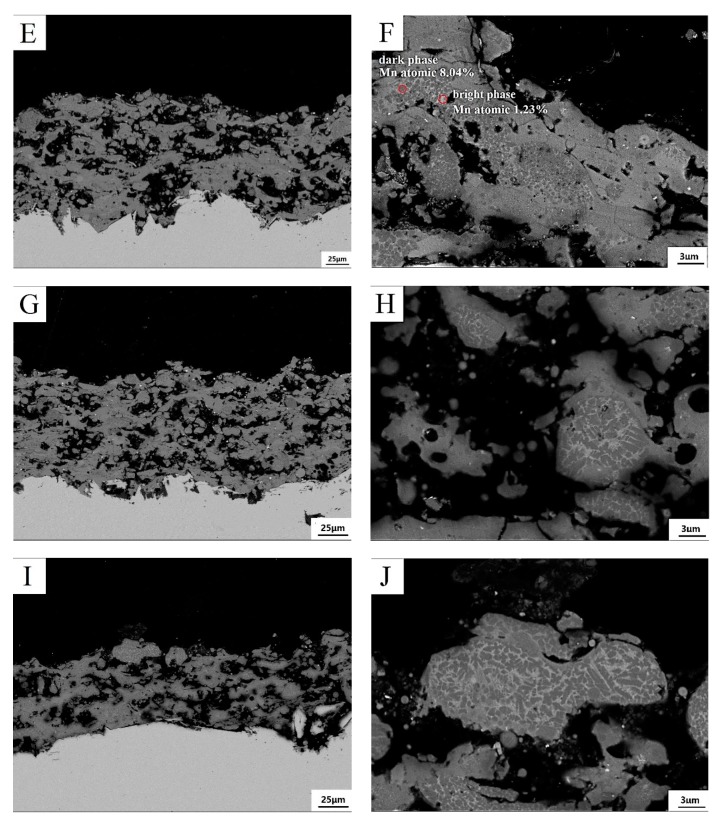
Low and higher magnification view of the cross section of coating with different composition. (**A**,**B**): 5MnO_x_/5CeO_2_/TiO_2_ coating; (**C**,**D**): 10MnO_x_/5CeO_2_/TiO_2_ coating; (**E**,**F**): 20MnO_x_/5CeO_2_/TiO_2_ coating; (**G**,**H**): 30MnO_x_/5CeO_2_/TiO_2_ coating; (**I**,**J**): 40MnO_x_/5CeO_2_/TiO_2_ coating.

Typically, plasma spray coatings are characterized by a layered microstructure consisting of large amount lamella [20]. Each lamella is formed by an individual molten and semi-molten particle impinging on the substrate. The cross-sectional morphology of the five coatings are shown in Figure 6. The result showed that the coating consisted of a large amount of fully-molten particles and some semi-molten particles. In the magnified SEM images, it can be easily found that the lamella in the coatings were composed of bright phase and dark phase. The size of these phases was of 1–2 micrometer, being too small to be accurately discerned by EDS. The chemical composition analysis by SEM only can give a rough estimation. The black phase was a manganese-rich phase and the white phase was rich in titanium. In the spraying process, the MnO_x_ underwent phase change because of the input heat of plasma jet. By comparing the XRD pattern of coatings, the formation of Mn_5_O_8_ and Mn_3_O_4_ in the five coatings and Mn_2_Ti in 40MnO_x_/5CeO_2_/TiO_2_ coating might account for the formation of such a Mn-rich phase.

### 3.3. Catalytic Activity of Coatings Deposited on Steel Plate

**Figure 7:**
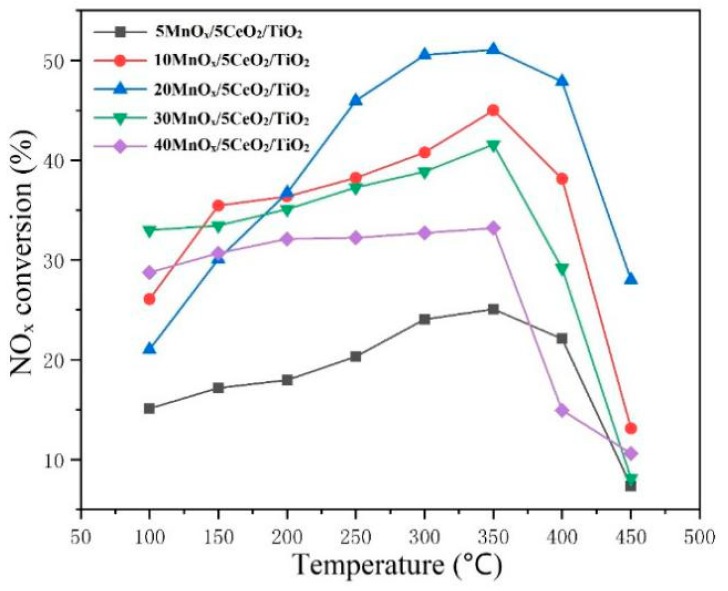
Nitrogen oxides (NO_x_) conversion of different MnO_x_/CeO_2_/TiO_2_ coatings.

**Figure 8:**
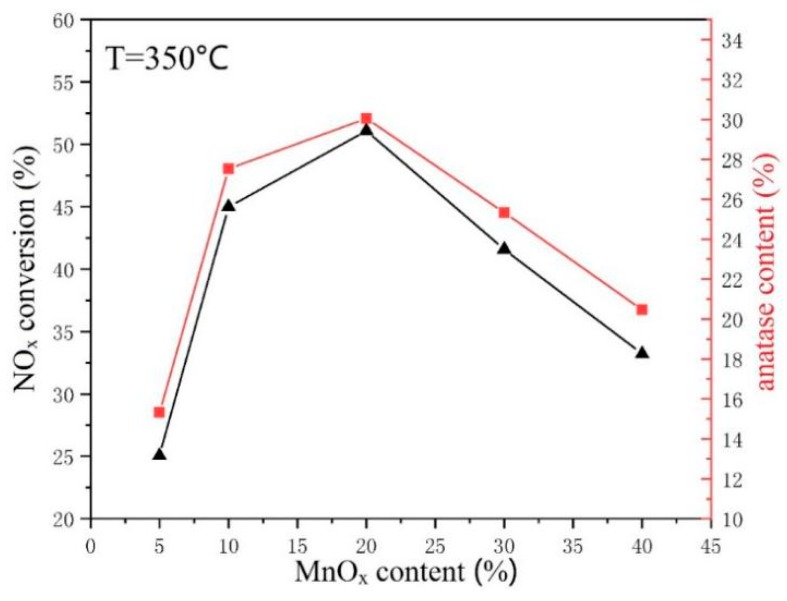
The dependence of the NO_x_ conversion efficiency on the MnO_x_ content and the anatase content.

The catalytic activity of the five coatings for SCR performance at different temperatures (ranged from 100–450 °C) is presented in Figure 7. The result showed that all the coating achieved their highest catalytic efficiency at a temperature of 350 °C, which is consistent with the data reported by A. Moscatelli [24]. Among all the coatings, the efficiency of the 20MnO_x_/5CeO_2_/TiO_2_ achieved 51.04% conversion of deNO_x_, which was more significant than the others. Curiously, the conversion efficiency of the coating was improved when the MnO_x_ loading was from 5–20%. As the loading of MnO_x_ continuously increased to 30%, 40%, the deNO_x_ efficiency decreased. The reason might be attributed to the variation of the anatase content in the coating. Some other works pointed out that MnO_x_ could have a positive effect with the support of anatase, in which the former could be dispersed more evenly. The presence of anatase in the catalyst can improve the catalytic performance effectively than rutile [3,25]. The relationship between the MnO_x_ content, anatase content and the NO_x_ conversion at 350 °C is summarized in Figure 8. It can be clearly seen that as the MnO_x_ content changed, the trend of catalytic efficiency of the coating consistently changed with the variation of the anatase content. When the fraction of MnO_x_ was 20%, the coating owned the highest anatase content and therefore the most powerful catalytic activity. Compared with traditionally powder-based Mn-Ce-Ti oxides catalyst, which achieved 90–100% NO_x_ conversion at the temperature of 100–220 °C [3,26,27], the coating catalyst on steel plate had a lower efficiency and higher reaction temperature. There are two possible reasons for this phenomenon. One was that powder-based materials had a much higher specific area than bulk materials, in which the former could contact intensively with the reaction gas. Moreover, compared with other Mn oxides like MnO_2_, Mn_2_O_3_, the Mn_3_O_4_, and Mn_5_O_8_ was generated in the sprayed coating, which had a relatively lower activity and might influence the react temperature consequently [28].

### 3.4. Catalytic Performance of the Coated Wire-Mesh Structure

**Figure 9:**
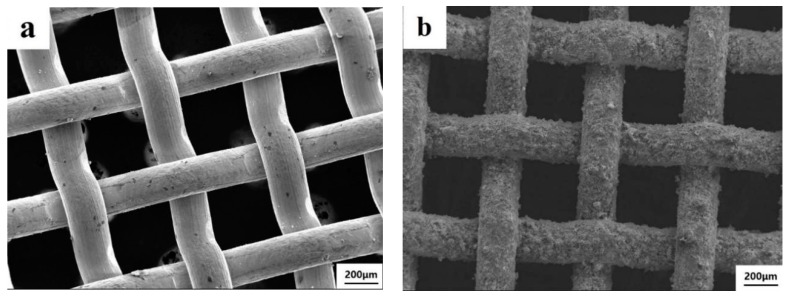
The morphology of wire-mesh before (**a**) and after (**b**) spraying.

**Figure 10:**
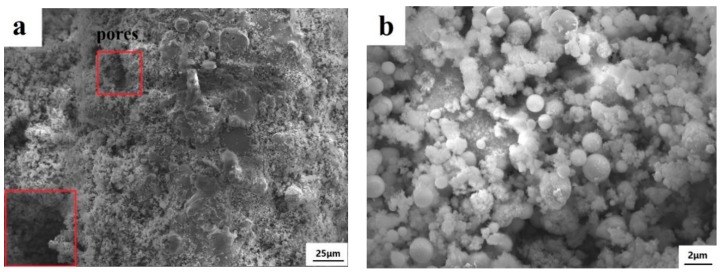
Surface morphology of the coating surface on wire-mesh (**a**) Low magnification, (**b**) Magnified observation.

**Figure 11:**
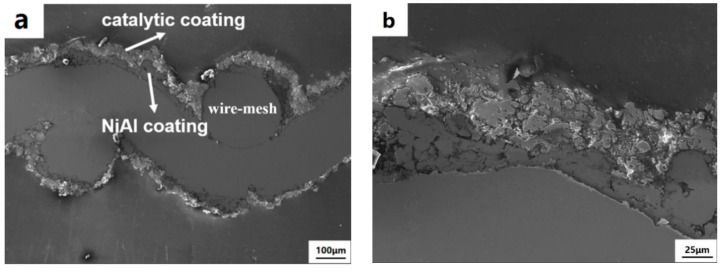
(**a**) Sectional morphology of the wire-mesh, (**b**) Enlarged SEM sectional morphology of the wire-mesh.

As the 20MnO_x_/5CeO_2_/TiO_2_ coating yields the highest deNO_x_ efficiency, this type of coating was selected and deposited on the wire-mesh substrate. The surface morphology of wire-mesh before and after spraying is shown in Figure 9. The magnified morphology of the coating surface and the sectional of coating is shown in Figure 10 and Figure 11, respectively. By comparing Figure 9a,b, it could be found that the surface of the wire-mesh was roughed after spraying. The sprayed catalytic coating consisted a lot of spherical particles and pores, as clearly shown in Figure 10. Those pores appeared on the coating surface formed by the irregular stacking of splats with different melting state. In Figure 10b, many spherical particles can also be observed on the coating surface, which might be attributed to the retention of the original nanoparticles. This implies that the coating was built up partly by the incomplete molten or semi-molten particles. Nevertheless, both these pores and spherical particles would enhance the roughness of coating surface, which more or less contributed to the improvement of the specific surface area [19]. From the sectional observation (Figure 11), the two layers can be clearly distinguished. The bottom layer was NiAl bond layer, which was only 10–20 μm thick and consisted of well-deformed particles. The top layer was 20MnO_x_/5CeO_2_/TiO_2_ coating.

**Figure 12:**
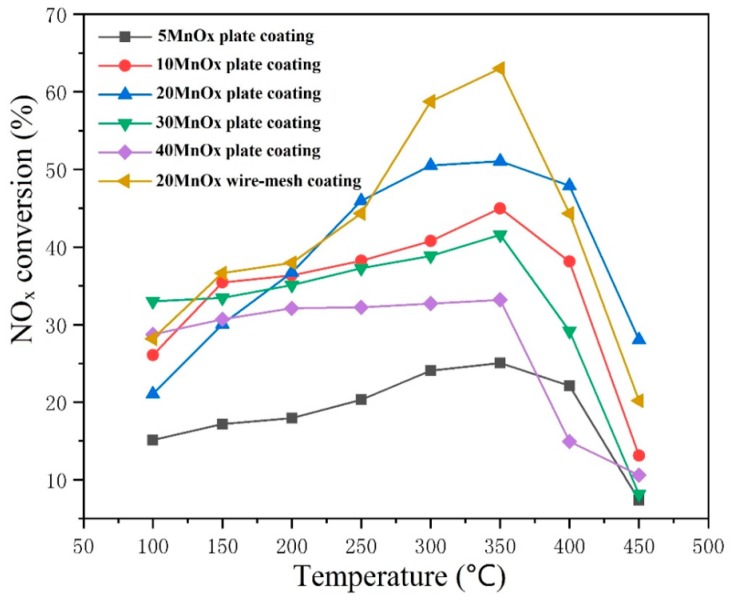
The activity of wire-mesh catalysts compared with stainless steel plates.

**Figure 13:**
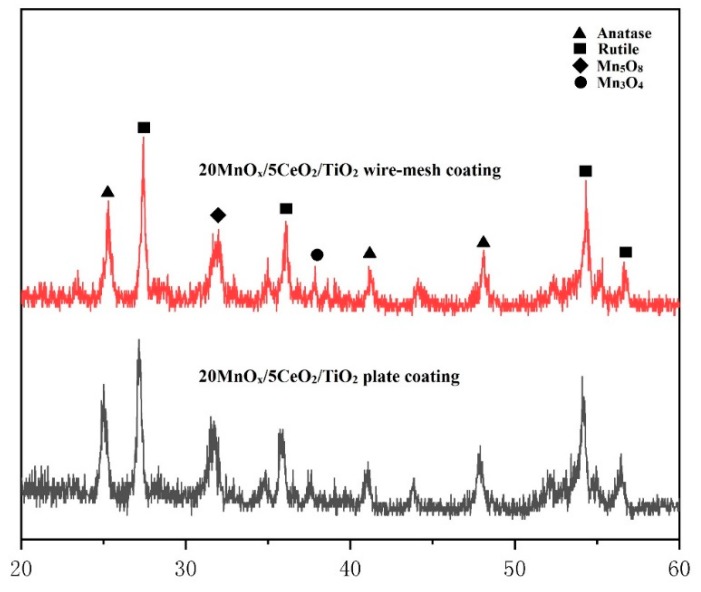
The XRD patterns of the wire-mesh coating compared with plate coating.

The result of the test on the catalytic activity of the wire-mesh catalysts as well as the steel plate samples are shown in Figure 12. It can be seen that the wire-mesh catalytic efficiency achieved 63% at 350 °C, which was much higher than the other steel plate. From the XRD patterns (Figure 13), no remarkable difference of the phase content can be found for the coating deposited on the steel plate substrate and wire-mesh substrate. The discrepancy in terms of the catalytic performance between the two catalytic coatings can be mainly attributed to their structural difference. The wire-mesh could take the advantage the surface-area-to-volume because of the net structure, which enhanced the catalyst load quantity [29]. In the case of the same GHSV, more catalyst had a longer time to contact with NO_x_, which increased the NO_x_ conversion rate further. Moreover, the dependence of catalytic performance of coating upon the testing temperature was different for two different substrates. We thought that the variation of substrate morphology might have a certain effect. When the coating was deposited on wire-mesh substrate with a curved surface, most of the sprayed particles was impacted obliquely, leading to an insufficient deformation at their impact. Some active components might be encapsulated so that they cannot work well at low temperature. As the temperature raised, a large number of the active component begins to function. When the temperature was further increased, these active components would rapidly lose their activity, resulting in a sudden decrease of conversion efficiency. Moreover, N_2_ selectivity is another standard to evaluate the performance of catalysts, which was influenced by the formation of NO_2_ and N_2_O in the reaction [22,30]. During the process of NH_3_-SCR, NO_2_ might be generated by the oxidization of NO (5) and (6), moreover NH_3_ might be excessively oxidized to N_2_O through the following reactions (6) and (7):

NO + O_2_ = NO_2_ (5)

4NH_3_ + 4NO + 3O_2_ = 4N_2_O + 6H_2_O (6)

4NH_3_ + 4NO_2_ + O_2_ = 4N_2_O + 6H_2_O (7)

**Figure 14:**
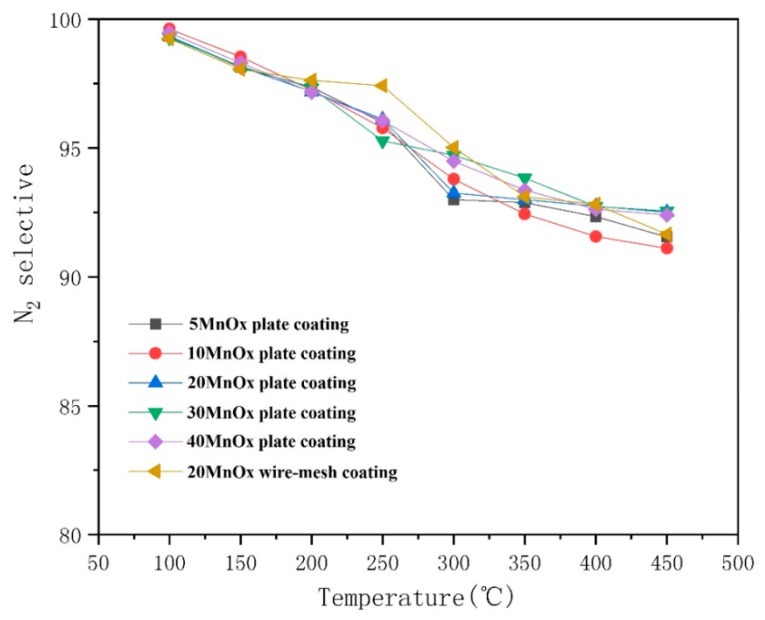
The nitrogen (N_2_) selectivity of different catalytic coatings.

The results of N_2_ selectivity calculated by Equation (4) is shown in Figure 14. It was found that the N_2_ selectivity of all the catalytic coatings were decreased as the temperature raised. The oxidization of NO would be promoted as temperature increased. Moreover, it was reported that N_2_O is mainly generated via the Eley–Rideal mechanism, which could also be improved as temperature raised [22,30]. These reasons might account for the reduction of N_2_ selectivity in the temperature range of 100–450 °C. Although the N_2_ selectivity was in a declining trend, it was more than 91% in the whole process, and it presented the same tendency among different coatings. This indicated that the plasma sprayed catalytic coating had a well selectivity and a good stability.

### 3.5. Stability of the Catalytic Performance

**Figure 15:**
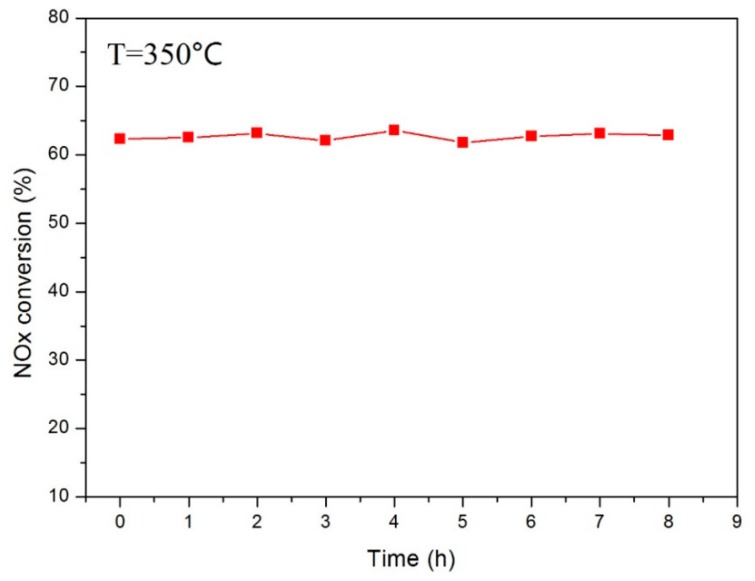
The activity of wire-mesh catalysts under continuously mixed gas.

To study the stability of the catalyst, the wire-mesh coating was tested under the continuously inputted mix gas circumstance at the temperature of 350 °C. The result of NO_x_ conversion is shown in Figure 15. It can be seen that the activity of the catalyst did not have an obvious change in the eight-h test, which indicated a relatively stable performance of the catalyst. The stability and the service life of the deposited catalytic coating in practical application still needs a further study.

## 4. Conclusions

In this work, different MnO_x_/CeO_2_/TiO_2_ coatings were firstly deposited on stainless steel plates by plasma spraying. The results showed that the original anatase phase was transformed to rutile to a certain degree. When the content of MnO_x_ was 20%, the remained anatase was higher than others. Under SCR test, the 20MnO_x_/5CeO_2_/TiO_2_ coating presented the highest catalytic activity, namely, 51.08% at 350 °C. Then, the 20MnO_x_/5CeO_2_/TiO_2_ coating was deposited on a wire-mesh substrate. Due to the complex structure of the wire-mesh, the maximum conversion efficiency of the coating on the wire-mesh substrate reached 63.07% at 350 °C. The plasma sprayed catalytic coating showed a valuable prospect in industrial SCR applications.

## Figures and Tables

**Table 1:** Parameters of plasma spraying with different coatings.

Coating Material	Power (kw)	Voltage (V)	Current (A)	Ar Flow Rate (SLPM)	Powder (g/min)	Spray Distance (mm)
NiAl	30	60	500	50	60	100
MnO_x_-CeO_2_-TiO_2_	16	40	400	40	30	100

**Table 2:** The content of anatase in the coating with different MnO_x_ content.

Coating Composition	TiO_2_ (Anatase) Content (wt %)
5MnO_x_/5CeO_2_/TiO_2_	14.53
10MnO_x_/5CeO_2_/TiO_2_	26.43
20MnO_x_/5CeO_2_/TiO_2_	28.75
30MnO_x_/5CeO_2_/TiO_2_	24.78
40MnO_x_/5CeO_2_/TiO_2_	16.98
